# Prognostic performance of the BAN-ADHF score in acute decompensated heart failure: a sub-analysis of the Spanish Heart Failure Registry (RICA)

**DOI:** 10.1007/s11739-026-04370-6

**Published:** 2026-08-07

**Authors:** A. Campos-Sáenz de Santamaría, F. Croset, J. Pérez-Santana, M. Sánchez-Marteles, J. Pérez-Silvestre, M. Montero-Pérez-Barquero, P. Llàcer, J. C. Trullàs, J. L. Morales-Rull, J. Casado, A. Pandey, J. Rubio-Gracia

**Affiliations:** 1 Faculty of Medicine, University of Zaragoza, Zaragoza, Spain; 2 Internal Medicine Department, Hospital Clínico Lozano Blesa, Avenue San Juan Bosco 15, 50009 Saragossa, Spain; 3 Aragon Health Research Institute (IIS Aragon), Saragossa, Spain; 4 Internal Medicine Department, Hospital Universitario Ramón y Cajal, IRYCIS, Madrid, Spain; 5 Department of Medicine and Medical Specialties, Facultad de Medicina y Ciencias de la Salud, Universidad de Alcalá, IRYCIS, Madrid, Spain; 6 Internal Medicine Department, Hospital Nuestra Señora Candelaria, Tenerife, Tenerife Spain; 7 Internal Medicine Department, Consorcio Hospital General Universitario Valencia, Valencia, Spain; 8 Internal Medicine Department, Hospital Reina Sofía, Córdoba, Spain; 9 Internal Medicine Department, Hospital d’Olot i comarcal de la Garrotxa, Girona, Spain; 10 Laboratori de Reparació i Regeneració Tissular (TR2Lab), Facultat de Medicina, Universitat de Vic, Universitat Central de Catalunya, Vic, Barcelona Spain; 11 Internal Medicine Department, Heart Failure Unit, Hospital Universitari Arnau de Villanova, Institut de Recerca Biomédica (IRBLleida), Lleida, Spain; 12 Internal Medicine Department, Hospital Universitario de Getafe, Madrid, Spain; 13 Division of Cardiology, Department of Internal Medicine, University of Texas Southwestern Medical Center, Dallas, TX USA

**Keywords:** Acute decompensated heart failure (ADHF), Heart failure (HF), Left ventricular ejection fraction (LVEF), Spanish Heart Failure Registry (RICA)

## Abstract

The BAN-ADHF score is a recently developed measure designed to predict low diuretic efficiency and adverse outcomes using eight simple routinely available clinical and laboratory variables. However, its performance in real-world settings remains understudied. This is a retrospective analysis using data from the Spanish Heart Failure Registry (RICA), a nationwide, prospective cohort of patients admitted for ADHF to internal medicine departments. Only patients with complete data to calculate the BAN-ADHF score were included. Patients were classified into low BAN-ADHF (< 12) and high BAN-ADHF (≥ 12) groups. The primary endpoint was all-cause mortality. Secondary endpoints included the composite of mortality and/or heart failure (HF) readmission and length of hospital stay. A total of 2110 were eligible. High BAN-ADHF patients (17.5%) were older, with a higher burden of comorbidities, worse functional class and a higher proportion of reduced left ventricular ejection fraction (LVEF). Additionally, high BAN-ADHF patients showed lower hemoglobin, sodium, and estimated glomerular filtration rate, together with higher levels of creatinine, potassium, CA125, and NT-proBNP. After a median follow-up of 363 days, all-cause mortality occurred in 30.3% of patients, predominantly in the high BAN-ADHF group (HR 1.75, 95% CI 1.46–2.08, *p* = 0.001). The composite endpoint occurred in 44.9% of patients and was more frequent in the high BAN-ADHF group (HR 1.47, 95% CI 1.24–1.76, *p* = 0.001). Moreover, the BAN-ADHF score was independently associated with longer hospital stays (*p* = 0.001). No interaction was found between LVEF and the BAN-ADHF categories. The BAN-ADHF score is a valuable prognostic tool for identifying ADHF patients at greater risk of mortality, rehospitalization, and longer hospitalization in a real-world setting. Its integration into routine care may help guide early therapeutic strategies and resource allocation.

**Graphical abstract:**

**Figure:**
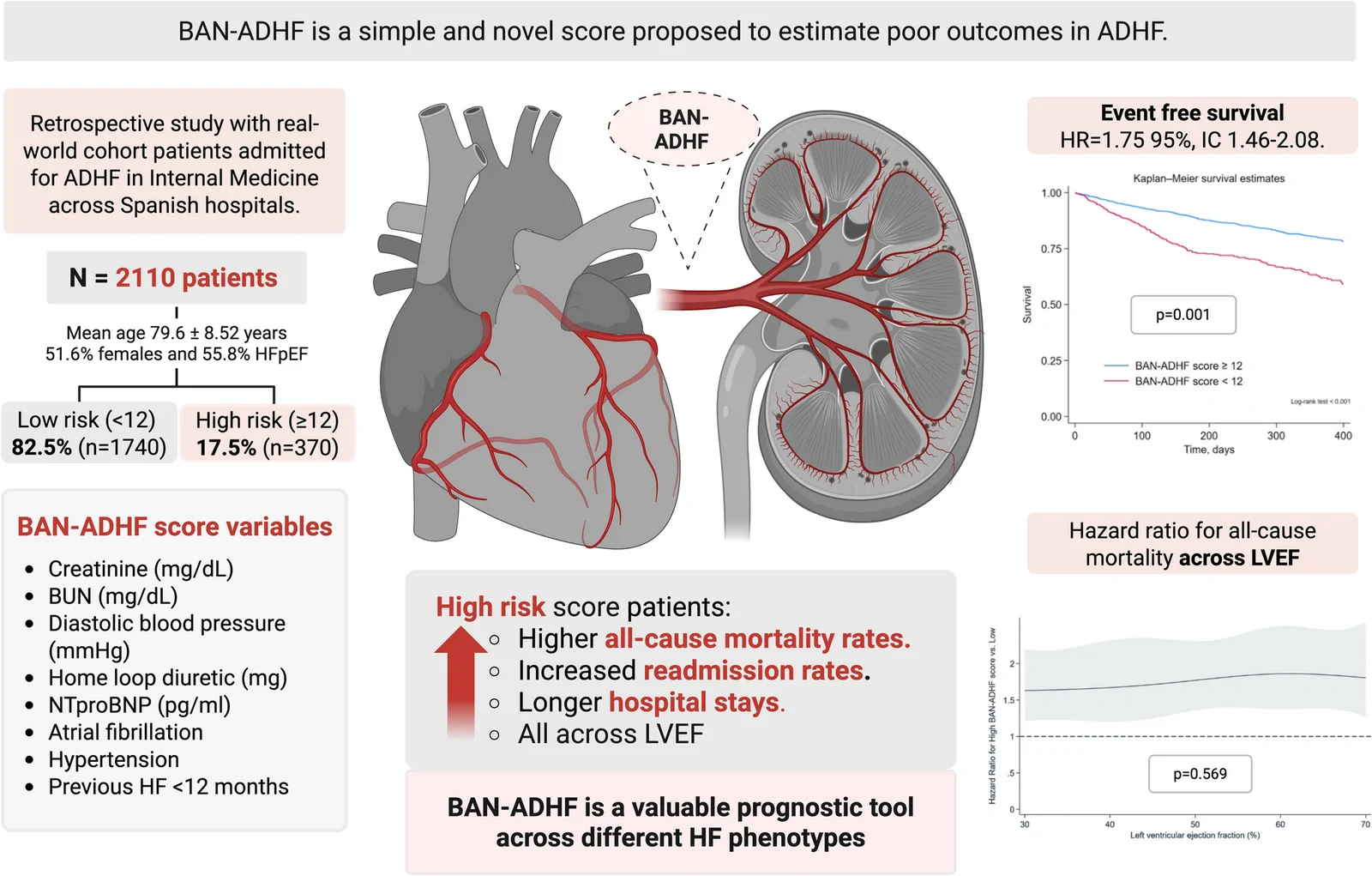

**Supplementary Information:**

The online version contains supplementary material available at https://doi.org/10.1007/s11739-026-04370-6.

## Introduction

In acute decompensated heart failure (ADHF), achieving an effective diuretic response is crucial to alleviate volume overload and improve outcomes [1, 2]. However, many patients are discharged with residual congestion [3], a key marker of poor prognosis. This condition may result from low diuretic efficiency or from suboptimal diuretic administration not guided by objective measures such as urinary sodium assessment [1]. Early identification of patients with low diuretic efficiency is essential to mitigate complications [4, 5].

**Fig. 1:**
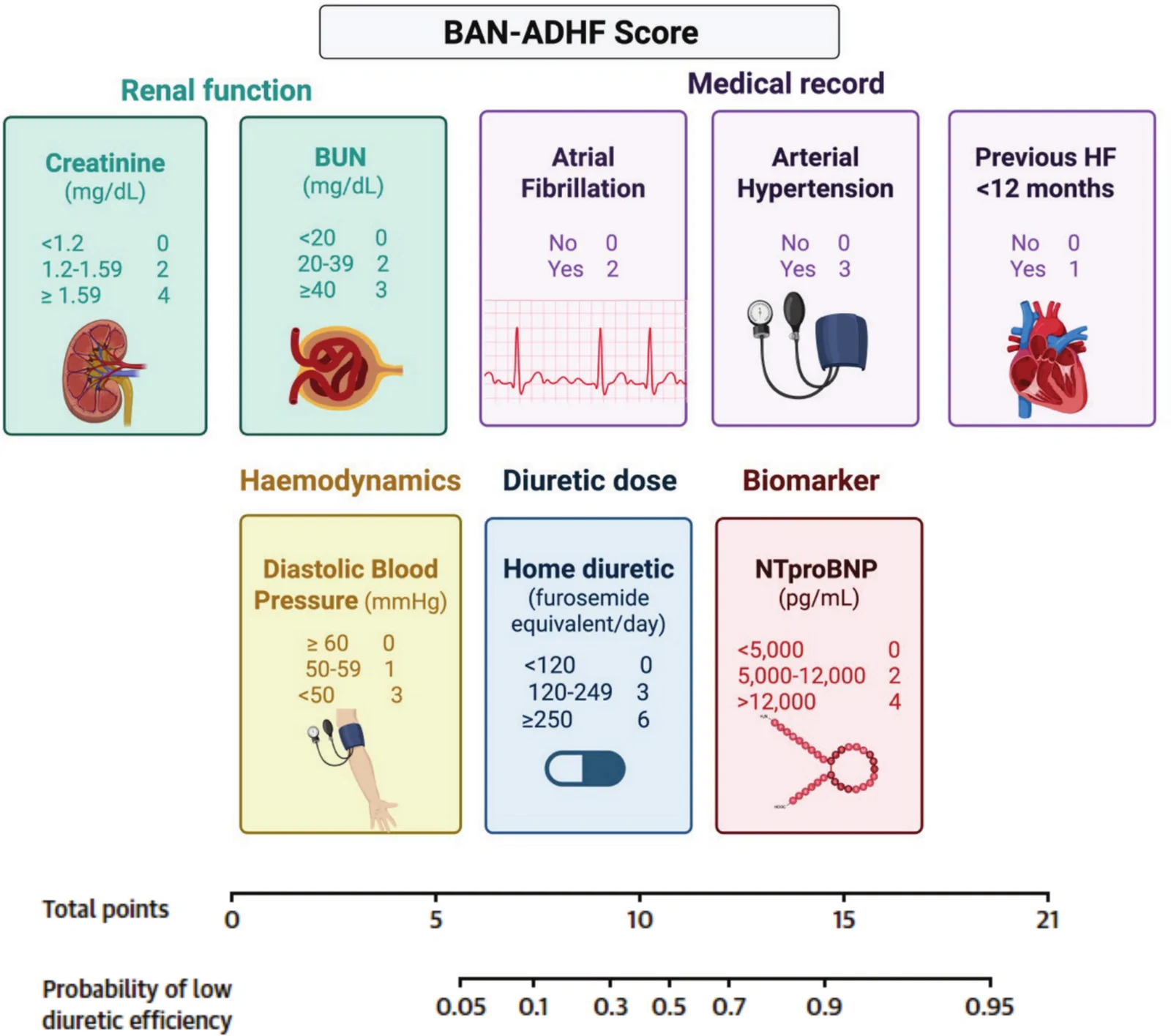
The BAN-ADHF Score proposed by Segar et al.

Segar et al. [6] recently introduced a machine learning-based model to predict low diuretic efficiency by using data from ROSE-AHF [7], CARRESS-HF [8], and ATHENA-HF [9] trials. This scoring tool was named “BAN-ADHF score” (Fig. 1), which summarizes the eight clinical and laboratory parameters included in the model. After its development, external validation was confirmed in the DOSE [10], ESCAPE [11], and GWTG-HF registry [12] cohorts, demonstrating an excellent predictive performance (C-index 0.92). In this context, patients with higher BAN-ADHF scores at admission showed worse outcomes, including longer hospital stays, reduced well-being, and higher in-hospital mortality.

The Spanish Heart Failure Registry (RICA) [13] is a nationwide, multicenter and prospective cohort that includes HF patients hospitalized in internal medicine departments. Unlike clinical trials, which often have strict inclusion criteria and a focus on specific subgroups, the RICA registry reflects a real-world population, encompassing a wide range of ejection fraction levels and comorbid conditions.

This study investigates whether the BAN-ADHF score provides additional prognostic information for predicting long-term all-cause mortality and heart failure readmissions, over and above conventional clinical risk factors, in a real-world population of patients admitted with acute decompensated HF.

## Methods

### Study design and patients

The present study is retrospective sub-analysis of patients included in the Spanish Heart Failure Registry (RICA), whose design has been described in detail elsewhere [13]. Briefly, the RICA cohort is a prospective multicentre registry that included patients consecutively admitted to 34 Spanish Internal Medicine Departments for ADHF from March 2008 to December 2020, who were discharged alive after the index ADHF episode that caused hospitalization. Follow-up visits were mandatory at 90 and 365 days after hospital discharge and subsequently, the patients’ vital status was subsequently reviewed annually. The RICA protocol was approved by the Ethics Committee of each participating center, and written informed consent was obtained from all participating patients.

### Patient selection and classification

**Fig. 2:**
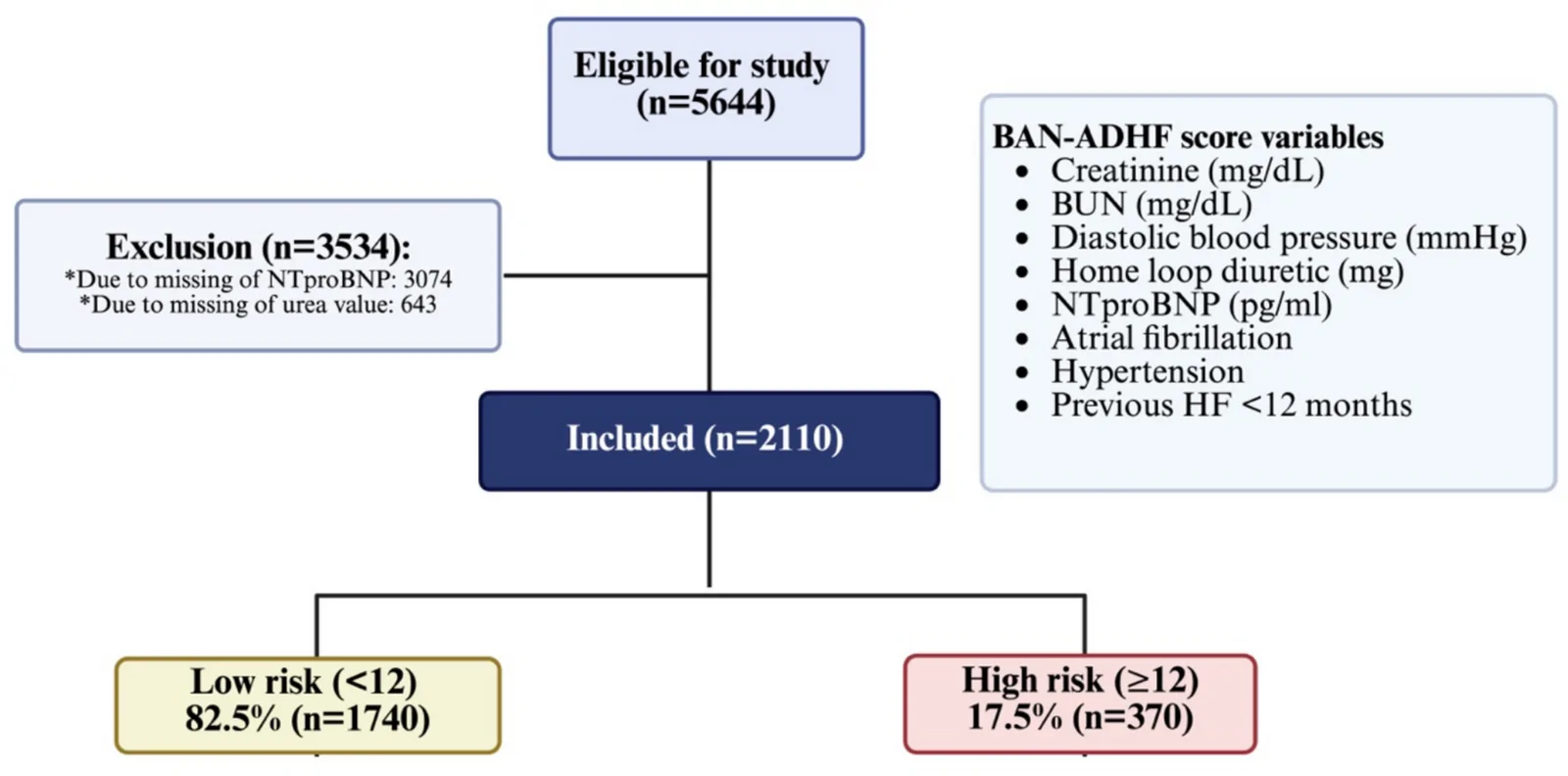
Flow Diagram of Patient Inclusion

The inclusion criteria of the RICA registry are adults (≥ 18 years) with a diagnosis of HF according to European guidelines, including both de novo and chronic HF, regardless of LVEF, etiology, comorbidities, or clinical phase (acute or stable). For the current study, only those patients with complete data to fulfill to BAN-ADHF score were included, as summarized in the flowchart (Fig. 2). The covariates included in the model are serum creatinine, blood urea nitrogen (BUN), diastolic blood pressure, home loop diuretic dose, NT-proBNP, atrial fibrillation, hypertension, and previous HF hospitalization within the preceding 12 months. In the RICA registry, serum creatinine, BUN, and NT-proBNP were measured at admission, diastolic blood pressure was recorded on initial presentation, and the remaining variables were abstracted from medical history and home medications. According to the BAN-ADHF score cut-off values proposed by Segar et al. [6], patients were classified into two groups: “Low BAN-ADHF” if the BAN-ADHF score was < 12 points on admission, and “High BAN-ADHF” if the BAN-ADHF score was ≥ 12 points on admission.

### Endpoints

The primary endpoint was all-cause mortality. Secondary endpoints included HF readmission, the composite of all-cause mortality and/or HF readmission and length of hospital stay. HF readmission was recorded individually by experienced investigators involved in patient inclusion and follow-up. It was defined as any unplanned HF decompensation requiring a hospital stay lasting more than 24 h and intravenous diuretic therapy. Information on all-cause mortality and HF readmissions was collected from the RICA registry, which prospectively tracks patients after hospital discharge. Follow-up visits were scheduled at 90 and 365 days, and thereafter, vital status was reviewed through medical records, and national death registries. Patients with incomplete follow-up were censored at the last known contact.

### Statistical analysis

Continuous variables are expressed as mean ± standard deviation or median [interquartile range], depending on normality assessed by the Kolmogorov–Smirnov test and normality plots. Categorical variables are shown as percentages with the total number of participants in parentheses. Baseline characteristics between the groups (BAN-ADHF score ≥ 12 vs. < 12) were compared using the *χ*^2^ test for categorical variables, and the t-test or Wilcoxon rank-sum test for continuous variables. Time-to-event analyses were performed using a multivariable Cox proportional hazards regression model. Risk estimates were presented as hazard ratios (HR) with 95% confidence intervals (CI). Variables included in the model were chosen based on those used in the multivariable analysis of the original study in which the BAN-ADHF score was first developed. The final multivariable model included the following covariates: age, sex, diabetes mellitus, systolic blood pressure, left ventricular ejection fraction (LVEF), plasma sodium levels, body mass index (BMI), and NYHA functional class. Potential collinearity among variables, including renal function measures such as creatinine, BUN, and eGFR, was assessed using correlation matrices and variance inflation factors (VIF), and no significant collinearity was observed that would compromise model stability. The proportional hazards assumption was tested using Schoenfeld residuals. To test the possible interaction between LVEF and the BAN-ADHF score categories (high BAN-ADHF vs. low BAN-ADHF) on prognosis outcomes, regression models were fitted for the endpoints, incorporating LVEF transformed by restricted cubic splines as a fixed effect, with patient identifier included as a random intercept. The restricted cubic splines were constructed using four knots for LVEF. Additionally, a multivariable linear regression analysis was performed to evaluate the association between the BAN-ADHF score and length of hospital stay. A two-sided p-value < 0.05 was considered statistically significant for all analyses. All analyses were conducted using STATA version 18.1.

## Results

### Baseline characteristics

Of the 5644 patients enrolled in the RICA Registry, 3534 were excluded due to missing variables required for calculating the BAN-ADHF scale, resulting in a final study sample of 2110 patients. Specifically, NT-proBNP and urea values were missing in 3074 and 643 patients, respectively.

Baseline characteristics of both included and excluded patients overall are shown in detail in Supplementary Material Table 1. Excluded patients were not stratified according to BAN-ADHF score, as the main reasons for exclusion were missing NT-proBNP and BUN, which area essential variables for score calculation and this would be misleading or result in inappropriate comparisons. Overall, both groups were comparable in terms of age, sex distribution, LVEF, comorbidities, and medical therapy. Some differences were noted: excluded patients had a slightly higher rate of recent hospitalizations, a worse functional status (higher NYHA class and lower Barthel index), and a greater prevalence of CKD and diabetes.

**Table 1:** Baseline characteristics stratified according to the BAN-ADHF score

Variables	Total population	Low-risk BAN-ADHF (< 12)	High-risk BAN-ADHF (≥ 12)	*p* value
Number of patients (%)	2110 (100%)	1740 (82.5%)	370 (17.5%)	**–**
*Demographics*				
Age (years)	79.6 ± 8	79 ± 8	81 ± 7	** < 0.001**
Female sex %	51.6% (1088)	53.4% (929)	43.2% (160)	** < 0.001**
*Heart failure characteristics*				
Left ventricular ejection fraction (%)	55 [22	55 [22]	50 [24]	** < 0.001**
Heart failure category (%)				** < 0.001**
HFpEF	55.8% (1177)	57.5% (1000)	47.3% (175)	
HFrEF	38.9% (821)	36.6% (638)	46.6% (172)	
HF hospitalizations < 12 months (%)	22.9% (483)	19.5% (339)	39.5% (146)	** < 0.001**
NYHA (%)				** < 0.001**
I	8.8% (194)	9.6% (167)	4.3% (16)	
II	53.7% (1133)	55.1% (958)	45.9% (170)	
III	33.4% (705)	31.2% (543)	42.7% (158)	
IV	3.2% (67)	2.7% (47)	5.7% (21)	
*Comorbidities*				
Barthel index (points)	95 [25]	95 [25]	90 [35]	** < 0.001**
Pfeiffer index (points)	1 [2]	1 [2]	1 [3]	**0.023**
Body mass index kg/m^2^	28.4 [7]	28.4 [7]	28.3 [6]	0.059
Atrial fibrillation %	55.1% (1162)	51.1% (889)	74.1% (274)	** < 0.001**
Hypertension %	87.4% (1844)	85.2% (1482)	97.8% (362)	** < 0.001**
Diabetes mellitus %	46.5% (981)	45.5% (791)	50.8% (188)	0.064
Dyslipidemia %	54.2% (1143)	52.9% (920)	60.3% (223)	**0.010**
Chronic kidney disease %	40.3% (850)	32.5% (565)	67.2% (248)	** < 0.001**
*Baseline treatment*				
ACEi %	37.1% (782)	39% (678)	28.4% (105)	** < 0.001**
ARB %	27.0% (569)	27.1% (471)	26.7% (99)	0.902
Beta-blockers %	70.9% (1496)	69.9% (1216)	75.4% (279)	**0.036**
Loop diuretic %	79.6% (1679)	79.1% (1376)	81.9% (303)	0.232
Loop diuretic dose—mg/day	55 [40]	40 [40]	80 [60]	** < 0.001**
Statin %	29.6% (624)	29.7% (516)	30% (111)	0.860
Thiazides %	15.4% (325)	14% (243)	21.9% (81)	** < 0.001**
MRA %	26.9% (567)	26.6% (462)	28.3% (105)	0.486
Digoxin %	23.4% (493)	28% (487)	22.4% (83)	0.608
*Vital signs*				
Systolic blood pressure mmHg	135 [33]	135 [33]	132 [34]	0.425
Diastolic blood pressure mmHg	72 [20]	73 [20]	70 [20]	** < 0.001**
Heart rate—rpm	82 [29]	87 [29]	83 [25]	**0.001**
*Laboratory*				
Hemoglobin (gr/dL)	12 [2.7]	12.2 [2.7]	11.6 [2.6]	** < 0.001**
Creatinine (mg/dL)	1.2 [0.6]	1.1 [0.5]	1.7 [0.7]	** < 0.001**
BUN (mg/dL)	61 [40]	56 [34]	81 [57]	** < 0.001**
Uric acid (mg/dL)	7.9 [3]	7.7 [3]	9.4 [3]	** < 0.001**
Sodium (mEq/L)	140 [5]	140 [5]	139 [6]	** < 0.001**
Potassium (mEq/L)	4.3 [0.8]	4.3 [0.7]	4.5 [0.8]	** < 0.001**
eGFR (mL/min)	55 [32]	59 [37]	37 [18]	** < 0.001**
NT-proBNP (pg/mL)	3589 [6554]	2962 [4379]	12,750 [13995]	** < 0.001**
CA125(U/mL)*	38 [76]	36 [72]	52 [78]	**0.034**
Troponin T (ng/mL)	0.03 [0.082]	0.027 [0.06]	0.06 [0.15]	** < 0.001**
High sensitivity troponin T (ng/mL)	37.5 [38.7]	35 [36.1]	48.4 [57.4]	**0.003**

*ACEi* angiotensin-converting enzyme inhibitors, *ARB* angiotensin receptor blockers, *BUN* blood urea nitrogen, *CA125* cancer antigen 125, *CHF* chronic heart failure, *eGFR* estimated glomerular filtration rate, *HFpEF* heart failure with preserved ejection fraction, *HFrEF* heart failure with reduced ejection fraction, *ICD* ischemic coronary disease, *iSGLT2* sodium–glucose co-transporter-2 inhibitors, *MRA* mineralocorticoid receptor antagonists, *NT-proBNP* N-terminal pro b-type natriuretic peptide, *NYHA* New York Heart Association

^*^CA125 was only available in 564 patients

Baseline characteristics overall and stratified according to the BAN-ADHF score are shown in detail in Table 1. The mean (SD) age of the participants was 79.6 ± 8.5 years, with a nearly equal sex distribution (51.6% were females) and predominant HFpEF (55.8%). The more prevalent comorbidities were hypertension (87.4%), atrial fibrillation (55.1%), diabetes (46.5%) and chronic kidney disease (40.3%), and 22.9% had been previously hospitalized for ADHF within the past 12 months.

Based on the BAN-ADHF score, 1740 patients (82.5%) were categorized as “low BAN-ADHF”, while 370 patients (17.5%) were classified as “high BAN-ADHF”. Compared to low BAN-ADHF patients, those in the high BAN-ADHF group were older, more frequently males, with a higher burden of comorbidities, worse functional class, more previous HF hospitalizations and a higher proportion of HFrEF. Additionally, high BAN-ADHF patients had worse analytical parameters, including lower hemoglobin, sodium and eGFR values (calculated by MDRD equation), and higher concentrations of creatinine, potassium, CA125 and NT-proBNP. Regarding HF-specific treatment, high BAN-ADHF patients were treated more frequently with beta-blockers and thiazide diuretics and received higher doses of loop diuretics.

### Clinical outcomes

**Fig. 3:**
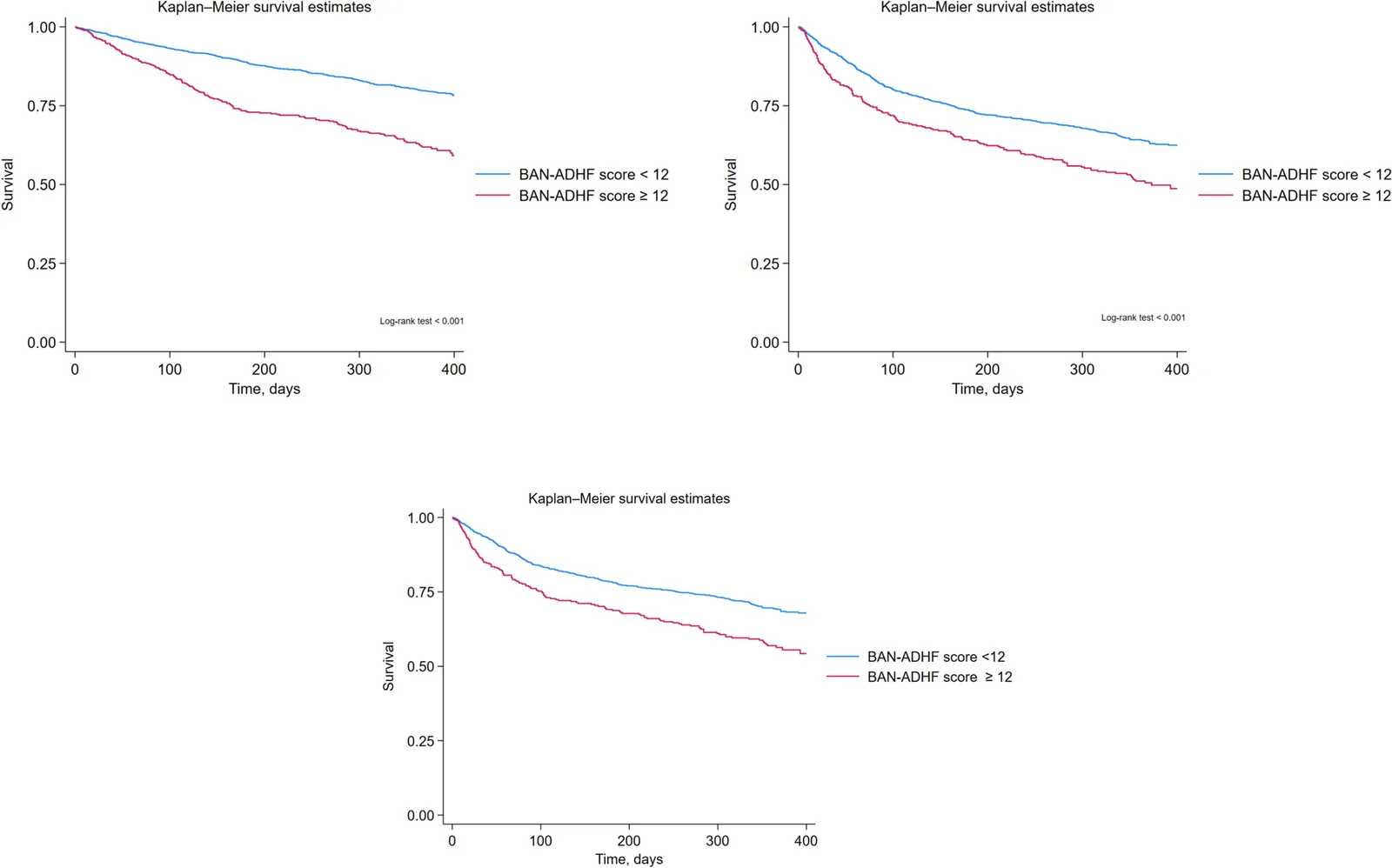
Kaplan–Meier survival curve by BAN-ADHF score for all-cause mortality (left), HF readmission (right) and the composite of both (below)

After a median follow-up of 353 days (IQR 250–395), a total of 640 patients (30.3%) died. In absolute terms, 472 deaths occurred in the low BAN-ADHF group (27.1% of that group) and 168 in the high BAN-ADHF group (45.4% of that group). Although the relative risk of death was significantly higher in patients with high BAN-ADHF scores, the majority of deaths occurred in the low BAN-ADHF group due to its larger size. 566 patients (26.8%) were readmitted for HF, and 949 patients (44.9%) reached the composite endpoint of all-cause mortality and/or HF readmission. Among patients who died, the median survival to death was 314 days (IQR 70–370). After multivariable Cox regression analysis, high BAN-ADHF patients showed a significant increase in both all-cause mortality (hazard ratio [HR] 1.75, 95% confidence interval [CI] 1.46–2.08, *p* = 0.001), HF readmission (HR 1.57, 95% CI 1.30–1.91, *p* = 0.001) and the composite endpoint (HR 1.48, 95% CI 1.24–1.76, *p* = 0.001) as compared with low BAN-ADHF patients (Tables 2 and 3). Kaplan–Meier survival curves for all-cause mortality, composite endpoint and HF readmission are presented in Fig. 3. This prognostic value was consistently observed in patients with and without CKD (*p* < 0.001 and *p* = 0.008, respectively) (Figs. 1 and 2 of Supplementary Material).

**Table 2:** Cox regression results for all-cause mortality

Variable	Model	HR	CI low	CI high	*p*-value
BAN-ADHF score ≥ 12	Univariable	1.86	1.56	2.21	< 0.001
	Multivariable	1.75	1.46	2.08	< 0.001
Age (years)	Univariable	1.03	1.02	1.04	< 0.001
	Multivariable	1.03	1.02	1.04	< 0.001
Sex (Male)	Univariable	0.93	0.79	1.09	0.38
	Multivariable	0.94	0.80	1.12	0.50
Systolic blood pressure (mmHg)	Univariable	1.00	0.99	1.00	0.18
	Multivariable	1.00	0.99	1.00	0.28
Diabetes mellitus	Univariable	0.94	0.80	1.10	0.43
	Multivariable	0.87	0.74	1.03	0.11
LVEF (%)	Univariable	1.00	0.99	1.00	0.21
	Multivariable	1.00	0.99	1.00	0.24
Plasma sodium (mmol/l)	Univariable	0.99	0.97	1.00	0.01
	Multivariable	0.99	0.98	1.00	0.04
BMI (kg/m^2^)	Univariable	0.97	0.96	0.99	0.00
	Multivariable	0.98	0.97	1.00	0.05
NYHA functional class	Univariable	1.00	0.99	1.01	0.89
	Multivariable	1.00	0.99	1.01	0.94

**Table 3:** Cox regression results for mortality and heart failure readmission

Variable	Model	HR	CI low	CI high	*p*-value
BAN-ADHF score ≥ 12	Univariable	1.48	1.25	1.76	< 0.001
	Multivariable	1.48	1.24	1.76	< 0.001
Age (years)	Univariable	1.01	0.99	1.01	0.23
	Multivariable	1.00	0.99	1.01	0.87
Sex (male)	Univariable	1.09	0.94	1.27	0.27
	Multivariable	1.08	0.92	1.26	0.36
Systolic blood pressure (mmHg)	Univariable	1.00	0.99	1.00	0.78
	Multivariable	1.00	0.99	1.00	0.94
Diabetes mellitus	Univariable	0.80	0.69	0.93	< 0.001
	Multivariable	0.79	0.68	0.93	< 0.001
LVEF (%)	Univariable	1.01	1.00	1.01	0.04
	Multivariable	1.01	1.00	1.01	0.05
Plasma sodium (mmol/l)	Univariable	0.99	0.98	1.00	0.04
	Multivariable	0.99	0.98	1.00	0.10
BMI (kg/m^2^)	Univariable	0.99	0.98	1.01	0.45
	Multivariable	0.99	0.98	1.01	0.27
NYHA functional class	Univariable	1.00	0.99	1.01	0.81
	Multivariable	1.00	0.99	1.01	0.92

**Fig. 4:**
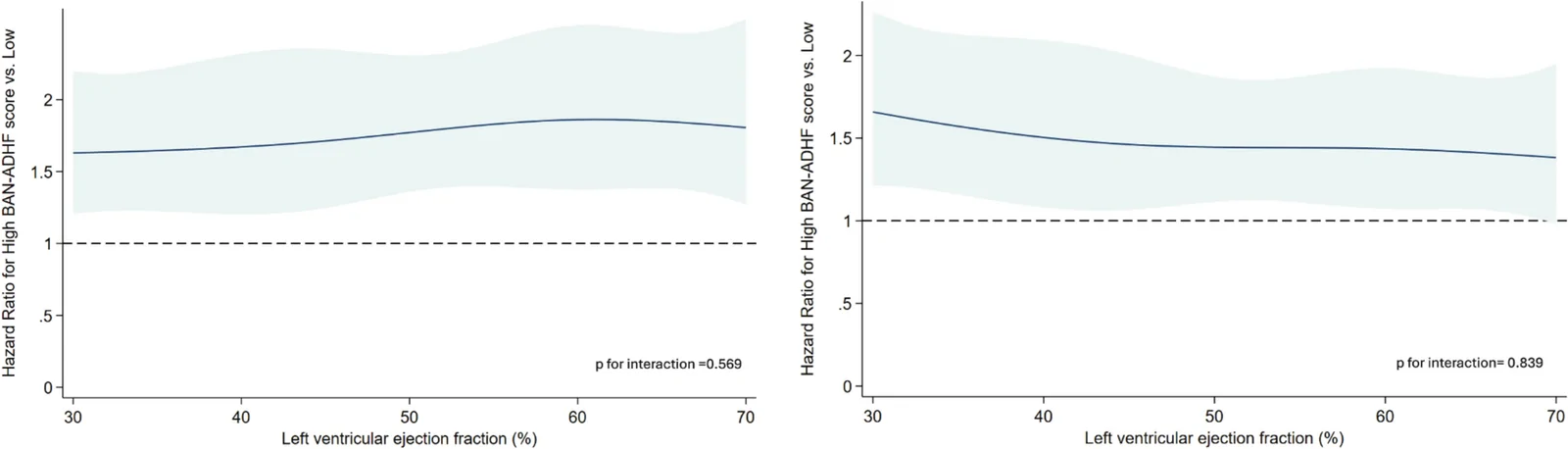
Hazard ratio for all-cause mortality (left) and composite endpoint (right) for high vs. low BAN-ADHF score categories across left ventricular ejection fraction

Furthermore, no interaction was observed between LVEF and the BAN-ADHF score categories (high vs. low) on both the primary endpoint of all-cause mortality (*p* for interaction = 0.569) and the combined endpoint (*p* for interaction 0.839) (Fig. 4).

In addition to the BAN-ADHF score, both univariable and multivariable Cox regression analyses identified key risk predictors for all-cause mortality and composite outcomes. Univariable analysis revealed that older age, lower plasma sodium levels, and lower BMI were associated with an increased risk of all-cause mortality. In contrast, diabetes mellitus was associated with a lower risk of the composite outcome, an effect that persisted in the multivariable analysis (Tables 2 and 3).

Finally, the association between the BAN-ADHF score and hospital length of stay was assessed using linear regression. The BAN-ADHF score was significantly associated with longer hospitalization (coefficient 0.30 days per 1-point increase in BAN-ADHF score; 95% CI 0.17–0.43; *p* < 0.001), indicating that patients with higher scores experienced longer hospital stays (Fig. 3 of Supplementary Material).

## Discussion

### Main findings

In this real-world cohort of patients hospitalized for ADHF from the Spanish RICA registry, the BAN-ADHF score is independently associated with post-discharge all-cause mortality, the composite endpoint of mortality and/or HF readmission, and longer length of hospital stay. Although the score was originally developed to predict diuretic efficiency and short-term in-hospital outcomes, our findings extend its prognostic utility to medium-term outcomes in a heterogeneous, unselected population treated predominantly in Internal Medicine departments. The predictive prognostic capacity of the BAN-ADHF score was analyzed in a real-world cohort of patients hospitalized for ADHF from the Spanish Heart Failure registry (RICA).

The main novelty of our study lies in the characteristics of the RICA population, which substantially differs from previous BAN-AHDF validation cohorts. Patients were older, had a high burden of comorbidities—including diabetes mellitus, CKD, hypertension and atrial fibrillation—and represented the full LVEF spectrum, with a particularly high prevalence of HFpEF. These features reflect cotemporary real-world AHD care and underscore the robustness of the BAN-ADHF score across diverse clinical contexts.

### Comparison with previous BAN-ADHF validation cohorts

Previous studies validating the BAN-ADHF score were mainly conducted in more selected populations, frequently younger, with lower comorbidity burden, and often managed in cardiology-led settings. In these cohorts, patients in the highest BAN-ADHF quartile exhibited poorer diuretic efficiency, greater congestion, and increased short-term mortality. Despite these differences, our results are remarkably consistent with prior evidence, showing a graded increase in mortality and adverse outcomes with higher BAN-ADHF scores [14, 15].

Importantly, the magnitude of risk observed in RICA may be influenced by population-level differences, including older age, higher prevalence of renal dysfunction, and broader inclusion criteria. These factors likely contribute to competing risks and may partially explain why, despite higher relative risk in the high BAN-ADHF group, most deaths in absolute terms occurred among patients with lower scores, reflecting the larger size of this subgroup. This observation highlights that while the BAN-ADHF score effectively identifies a high-risk phenotype, a substantial proportion of events still arise from patients traditionally considered at lower risk.

The persistence of the prognostic value of the BAN-ADHF score in a cohort with advanced age and substantial comorbidity burden supports the central role of congestion, renal dysfunction, and impaired natriuretic response in determining outcomes in ADHF. BAN-ADHF score originally conceived to capture diuretic efficiency retains prognostic significance in patients with multiple competing risks suggests that diuretic resistance and residual congestion remain pivotal drivers of rehospitalization and mortality, irrespective of HF phenotype. This finding is particularly relevant in HFpEF, a condition characterized by lower neurohormonal activation, different congestion patterns, less pronounced elevations of natriuretic peptides [17, 18] and decline in glomerular filtration primarily driven by venous congestion rather than renal hypoperfusion [19–21]. Despite these pathophysiological differences, the BAN-ADHF score maintained its discriminatory capacity across the entire LVEF spectrum, reinforcing its applicability beyond traditional HFrEF-focused risk models.

Indirect markers observed in our cohort further support a link between high BAN-ADHF scores and diuretic resistance. Higher loop diuretic doses, more frequent use of thiazide diuretics, lower hemoglobin levels, and elevated CA125 concentrations in patients with high scores suggest more severe congestion and impaired renal handling of sodium and water. The combination of diuretics may reflect some degree of diuretic resistance and an attempt to enhance decongestion through sequential nephron blockade [25, 26]. Although these associations are observational and hypothesis-generating, they align with previous evidence identifying CA125 and anemia as markers of congestive nephropathy and reduced diuretic efficiency [26, 27].

### Clinical implications

From a clinical perspective, the BAN-ADHF score offers a practical tool for early risk stratification in hospitalized ADHF patients. Unlike more complex prognostic models, such as BCN Bio-HF or MAGGIC score [23, 24], it relies on eight easily available variables and can be applied at the bedside, particularly in Internal Medicine settings where patients are older and clinically more complex. Early identification of patients at high risk of and adverse outcomes may facilitate more intensive monitoring, timely escalation of decongestive strategies, and optimized allocation of hospital resources [29].

Recent post-hoc analyses from randomized trials, such as CLOROTIC [30] and ADVOR [31], further reinforce the external validity of the BAN-ADHF score. These studies demonstrated that patients with high scores exhibit impaired diuretic and natriuretic responses and worse congestion trajectories, while also showing that sequential nephron blockade strategies can improve decongestion irrespective of baseline risk. Together with our findings, this evidence supports the role of BAN-ADHF not only as a prognostic marker but also as a potential guide for individualized decongestive therapy.

Additionally, the association between higher BAN-ADHF scores and longer length of hospital stay suggests that the score may help identify patients with greater healthcare resource utilization. This feature is particularly relevant in contemporary HF care, where reliable predictors of hospitalization burden remain limited.

## Limitations

Our study has several limitations. First, this study represents a retrospective sub-analysis of a prospective, multicenter registry (RICA). As such, although the registry itself was designed prospectively, some variables were collected retrospectively, which may limit causal inference. Additionally, only patients with complete data for the BAN-ADHF score were included, which could introduce selection bias. Second, the retrospective nature of this sub-analysis also raises the possibility of unmeasured confounding, and missing data may have influenced the results despite the registry’s overall completeness. It should be noted that excluded patients had a higher rate of recent hospitalizations, worse functional status (higher NYHA class and lower Barthel index), and a greater prevalence of chronic kidney disease and diabetes. These differences may introduce selection bias and could limit the external validity of our findings, as the study population may not fully represent all patients with acute HF. Furthermore, the predominance of low BAN-ADHF score among included patients could influence the results. Third, the score’s reliance on renal function parameters (creatinine and blood urea nitrogen levels) may reduce its utility in patients with severe kidney disease. Fourth, although both acetazolamide [32] and SGLT2 inhibitors [33] have been shown to enhance diuretic efficiency when used alongside loop diuretics, these treatments were not included in the present registry. Fifth, our record did not include serum albumin, which may have added prognostic information that could improve risk stratification. Sixth, data on in-hospital mortality and renal replacement therapy were unavailable, hindering a direct evaluation of BAN-ADHF on acute in-hospital outcomes. Although the study included a diverse patient population, the findings need validation in prospective cohorts and across different clinical settings and HF phenotypes to confirm their applicability. Additionally, the RICA registry did not systematically collect direct in-hospital measures of diuretic response, such as urinary sodium excretion, urine output per diuretic dose, or weight loss during hospitalization. Therefore, we were unable to compare the prognostic performance of the BAN-ADHF score with objective indicators of diuretic resistance assessed during the index admission. Future prospective studies should address this limitation and directly compare these approaches.

## Conclusions

This study explored the prognostic value of the BAN-ADHF score in a real-world cohort of patients hospitalized with ADHF from the RICA registry. Higher BAN-ADHF scores are associated with higher all-cause mortality, HF readmissions and longer hospital stays.

## Supplementary Information

Below is the link to the electronic supplementary material.

Supplementary file1 (DOCX 234 KB)

## Abbreviations

ADHF: Acute decompensated heart failure
BUN: Blood urea nitrogen
BMI: Body mass index
CA125: Cancer antigen 125
CKD: Chronic kidney disease
CI: Confidence interval
eGFR: Estimated glomerular filtration
HR: Hazard ratio
HF: Heart failure
HFpEF: Heart failure with preserved ejection fraction
HFrEF: Heart failure with reduced ejection fraction
IQR: Interquartile range
LVEF: Left ventricular ejection fraction
NT-proBNP: N-terminal pro b-type natriuretic peptide
NYHA: New York Heart Association
RICA: Spanish Heart Failure Registry
