# Supplementary material for: Prognostic performance of the BAN-ADHF score in acute decompensated heart failure: a sub-analysis of the Spanish Heart Failure Registry (RICA)
Source: Intern Emerg Med. 2026 Aug 7;21(6):2029–40. doi: 10.1007/s11739-026-04370-6 (PMC13582238; doi:10.1007/s11739-026-04370-6)
Supplement: Supplementary file 1 [file 11739_2026_4370_MOESM1_ESM.docx]

| **Variables** | **Included** | **Excluded** | **p value** |
| --- | --- | --- | --- |
| Number of patients | 2110 | 3534 | **-** |
| **Demographics** | | | |
| Age (years) | 79.6 ± 8 | 79.7±8 | 0.467 |
| Female sex | 51.6% (1088) | 55.6% (1966) | 0.668 |
| **Heart failure characteristics** | | | |
| Left Ventricular Ejection Fraction (%) | 55 [22] | 55 [15] | 0.920 |
| Heart failure category   - HFpEF - HFrEF | 55.8% (1177)  38.9% (821) | 58.5% (2068)  41.5% (1466) | 0.889 |
| HF hospitalizations <12 months | 22.9% (483) | 27.2% (963) | **<0.001** |
| NYHA   - I - II - III - IV | 8.8% (194)  53.7% (1133)  33.4% (705)  3.2% (67) | 6.1% (213)  35.8% (1267)  55.7% (1975)  2.2% (70) | **<0.001** |
| **Comorbidities** | | | |
| Barthel index | 95 [25] | 80 [24] | **<0.001** |
| Pfeiffer index | 1.5 [2] | 1.4 [2] | 0.138 |
| Body mass index | 28.4 [7] | 29.3 [8] | 0.947 |
| Atrial fibrillation | 55.1% (1162) | 54.1% (1914) | 0.170 |
| Hypertension | 87.4% (1844) | 90.6% (3204) | 0.924 |
| Diabetes mellitus | 46.5% (981) | 49.2% (1740) | **0.028** |
| Dyslipidaemia | 54.2% (1143) | 53.5% (1891) | 0.730 |
| Chronic Kidney Disease | 40.3% (850) | 56.9% (2011) | **0.034** |
| **Baseline treatment** | | | |
| ACEi | 37.1% (782) | 41.3% (1462) | 0.126 |
| ARB | 27.0% (569) | 28.7% (1016) | 0.439 |
| Beta-blockers | 70.9% (1496) | 70.9% (2506) | 0.158 |
| Loop diuretic | 79.6% (1679) | 78.2% (2766) | 0.243 |
| Loop diuretic dose (mg) | 55 [40] | 40 [40] | 0.113 |
| Statin | 29.6% (624) | 32.3% (1142) | 0.125 |
| Thiazides | 15.4% (325) | 16.5% (585) | 0.887 |
| MRA | 26.9% (567) | 22.4% (792) | **<0.001** |
| Digoxin | 23.4% (493) | 20.8% (735) | **0.005** |
| **Vital signs** | | | |
| Systolic blood pressure | 135 [33] | 135 [31] | 0.213 |
| Diastolic blood pressure | 72 [20] | 73 [22] | 0.743 |
| Heart rate (rpm) | 82 [29] | 87 [29] | 0.607 |
| **Laboratory** | | | |
| Haemoglobin (gr/dL) | 12 [2.7] | 12.1 [2.7] | 0.009 |
| Creatinine (mg/dL) | 1.2 [0.6] | 1.1 [0.6] | 0.356 |
| Uric acid | 7.9 [3] | 7.8 [3] | 0.009 |
| Sodium (mEq/L) | 140 [5] | 139 [6] | **<0.001** |
| Potassium (mEq/L) | 4.3 [0.8] | 4.3 [0.7] | 0.129 |
| eGFR (mL/min) | 55 [32] | 56 [35] | 0.248 |
| NT-proBNP (pg/ml) | 3589 [6554] | 4768 [6521] | 0.515 |
| CA 125(U/mL)* | 38 [76] | 55 [85] | 0.079 |

**Table 1. Differences between included and excluded patients.**

**Abbreviations**: ACEi: angiotensin-converting enzyme inhibitors; ARB: angiotensin receptor blockers; BUN: blood urea nitrogen; CA125: cancer antigen 125; CHF: chronic heart failure; eGFR: estimated glomerular filtration rate HFpEF: heart failure with preserved ejection fraction; HFrEF: heart failure with reduced ejection fraction ICD: ischaemic coronary disease; iSGLT2: sodium-glucose co-transporter-2 inhibitors; MRA: mineralocorticoid receptor antagonists; NT-proBNP: N-terminal pro b-type natriuretic peptide. *CA125 was only available in 564 patients.

**Figure 1.** Kaplan-Meier survival curve by BAN-ADHF score categories for composite end-point in patients with chronic kidney disease (CKD: eGFR < 60 mL/min/1.73m).

**Figure 2.** Kaplan-Meier survival curve by BAN-ADHF score categories for composite end-point in patients without chronic kidney disease (eGFR > 60 mL/min/1.73m).

**Figure 3.** Length of stay (days) across BAN-ADHF score.
